# In‐Flight Deterioration Occurs Early in Aeromedical Trauma Patients

**DOI:** 10.1111/1742-6723.70140

**Published:** 2025-09-11

**Authors:** Benjamin Powell, Susanna Cramb

**Affiliations:** ^1^ Retrieval Services Queensland Queensland Health Brisbane Queensland Australia; ^2^ LifeFlight Medical Brisbane Queensland Australia; ^3^ Faculty of Health, Medicine and Behavioural Sciences The University of Queensland Brisbane Queensland Australia; ^4^ Australian Centre for Health Services Innovation School of Public Health & Social Work, Queensland University of Technology Brisbane Queensland Australia; ^5^ Jamieson Trauma Institute Brisbane Queensland Australia

**Keywords:** aeromedical retrieval, pre‐hospital and retrieval medicine (PHRM), trauma

## Abstract

Reliably defining the risk of adverse in‐flight events in aeromedical trauma patients could enable more informed pre‐departure treatment and guide central asset allocation to achieve better system‐level outcomes. Unfortunately, the current literature base specifically examining the in‐flight period is sparse. Flight duration is often considered a proxy for the risk of in‐flight deterioration; however, there is limited data to support this commonly held assumption. This paper examines the association between flight duration and the risk of in‐flight deterioration in aeromedical trauma patients. A total of 2927 cases of aeromedical transport for acute trauma were retrospectively examined, and the time to first hypotension was recorded. Cases were categorised as either primary or inter‐hospital transfer retrievals. Cases were also subclassified as being a primary Traumatic Brain Injury or not based on several criteria, including initial GCS. The median time to hypotension was 11.5 min overall, 10 min in primary retrieval cases, and 15 min in inter‐hospital transfer cases (*p* = 0.049). Notably, after approximately 50 min, a significant plateau in cumulative risk was observed. Isolated TBI cases had a significantly higher overall rate of in‐flight hypotension, at 39.5% compared to 9.2%. Overall, this paper supports the physiologically plausible assumption that longer aeromedical transfer times are associated with an increased risk of deterioration during flight. It also demonstrates that deterioration tends to occur early in flight, raising questions as to why this might occur.

## 1 Introduction

In trauma patients, prolonged pre‐hospital ground transport times negatively affect morbidity and mortality [1, 2]. This is often assumed to translate to the aeromedical field and other markers of patient outcome, including the risk of in‐flight deterioration. However, there is limited high‐quality evidence examining how the duration of aeromedical retrieval affects the rate of in‐flight deterioration in adult trauma patients. As such, it is unclear if this commonly held assumption is correct.

In the Queensland aeromedical environment, long‐distance transfers are a common occurrence. The state covers 1.7 million square kilometres, [3] with the statewide retrieval service, Retrieval Services Queensland, routinely coordinating both short‐distance transfers of less than 20 min duration and long‐distance transfers of over 1000 km. As such, the relationship between transport time and the risk of deterioration is particularly important for the region.

The ability to anticipate in‐flight deterioration has several practical implications, including contributing to decision‐making regarding pre‐departure treatment. Responding to deterioration in the flight environment is significantly more challenging than on the ground, secondary to space limitations, movement restrictions within the cabin, reduced ambient lighting, reduced care provider numbers, equipment limitations, the effects of turbulence and altitude, and several other aviation factors [4, 5, 6, 7]. As such, determining who will deteriorate in flight and providing interventions to prevent this before take‐off is considered best practice. Currently, this is largely a gestalt‐based process with minimal supporting evidence, and prior work showing that hospital‐based trauma scores are of limited utility [8].

Determining cases at risk of in‐flight deterioration also has the potential to improve resource allocation. From a central coordination and asset allocation perspective, long‐distance transfers require a team to focus on a single patient for the majority of their shift, resulting in reduced assets and clinicians available for the remainder of the region. This can result in other patients having their transfers delayed and local doctors being tasked with caring for critically ill patients for an extended period. As such, being able to determine which patients are likely to deteriorate during their transport phase is of clinical importance and could improve resource utilisation. For example, if a patient is deemed to be low risk despite a long‐distance transfer, this might allow a doctor to be tasked with another patient with a higher risk of deterioration.

Despite all of this, the relationship between transfer time and the risk of in‐flight deterioration remains undetermined. It is also unclear if the length of retrieval is a dominant factor in the risk for in‐flight deterioration. Our prior work has demonstrated the difficulties in predicting in‐flight hypotension and the need for critical care interventions [8, 9]. This paper examines the relationship between the duration of transfer and the risk of in‐flight deterioration in aeromedical trauma patients.

## 2 Methods

Following ethics approval from the Darling Downs Hospital and Health Service Ethics Department (HREC/2022/QTDD/87421), a retrospective case analysis was conducted of all trauma patients transported on an aeromedical platform by a LifeFlight Medical doctor from January 1, 2020, to December 31, 2022. Data included the precise flight start and end time, as well as the time of first in‐flight hypotension. A credentialled senior aeromedical retrieval specialist reviewed all cases.

Cases were included if

- The primary diagnosis requiring aeromedical transport was acute trauma occurring within 24 h prior, as determined by senior retrieval clinician review of the written case notes.
- The patient underwent aeromedical transport under the care of a LifeFlight Medical doctor.
- The patient was at least 16 years old.
- Relevant data points were reliably and legibly recorded.

Cases were excluded if:

- Burns were the primary/sole injury.
- The patient was known to be pregnant.
- Transport occurred for a delayed complication of trauma, for example, cardiothoracic surgical management of delayed empyema following thoracostomy.
- The documentation was illegible or did not meet the minimum data set.

Transfer time was recorded in minutes and defined as the total time from documented aeromedical platform take‐off to arrival at the receiving facility or airport. For the purposes of this paper, “in‐flight” refers to the period between take‐off and landing. Timings for these calculations were derived from clinician‐recorded data, which are mandatory fields in the LifeFlight electronic notes system.

Cases were categorised as general trauma or isolated Traumatic Brain Injury (TBI). To be included in the TBI cohort, the following criteria were required:

- Clinical notes indicated an isolated head injury or trauma in which a head injury was the primary and most significant component requiring care, without other injuries requiring specific therapy that might complicate clinical decision‐making.
- Glasgow Coma Score (GCS) less than 14 (mild traumatic brain injuries excluded).

The outcome of interest was in‐flight hypotension. For general cases, this was defined as a mean arterial blood pressure (MAP) of less than 65 mmHg, and for TBI, a MAP of less than 80 mmHg.

Descriptive analyses of flight times and times to hypotension examined the median, interquartile range (IQR), and range; other continuous variables were considered only in terms of the median and IQR. Pearson's chi‐squared tests were used to compare median times across categorical variables, likewise for proportions. All analyses were conducted by transfer type—primary, Inter‐Hospital Transfer (IHT), or all combined. The Nelson‐Aalen cumulative risk curve was used to assess the accumulated risk of hypotension during a flight, by transfer type. Retrievals where the time to hypotension was missing were excluded.

Two sensitivity analyses were conducted. First, due to the potential unreliability of blood pressure monitoring immediately after departure and prior to landing, all hypotension cases occurring in‐flight within 5 min of flight departure or arrival were treated as non‐hypotension cases. Second, due to the potential for vasoactive drugs to influence haemodynamics, all cases that continued using vasopressors during flight were excluded. Nelson‐Aalen cumulative risk curves were then recalculated for each scenario, including by transfer type.

All analyses were conducted in Stata v18.0 MP (StataCorp, College Station, TX, USA).

## 3 Results

Once the selection criteria were applied, 2927 episodes of aeromedical transport for acute trauma were identified during the study period (Table 1). Only four cases were excluded due to absent or unreliable time data. There were similar case numbers between primary and IHT retrievals, at 1418 and 1509, respectively. 74% of all people were male, with a consistent median age of 44 years. The majority of primary cases were rotary wing (99%), as opposed to 69% in IHTs. The median flight time in IHTs was significantly longer, at 55 min, compared to 31 min for primary cases (*p* < 0.001). Despite this, there was a minimal difference in median time to hypotension, at 10 min for primary cases and 15 min for IHTs (*p* = 0.049). Significantly more IHT cases met the TBI definition (14%) compared to primary retrievals (6%), with 10% of the overall cohort meeting the isolated TBI definition (*p* < 0.001).

**TABLE 1:** Characteristics of included patients.

	Primary	Inter‐hospital	All
Median (IQR)			
Age	44 (27, 58)	44 (26, 62)	44 (27, 60)
SBP	128 (114, 142)	127 (114, 141)	127 (114, 142)
MAP	86 (77, 96)	84 (75, 94)	85 (76, 95)
Numbers (%)			
Male	1040 (73%)	1121 (74%)	2161 (74%)
Traumatic brain injury^a^	78 (6%)	210 (14%)	288 (10%)
Helicopter	1405 (99%)	1038 (69%)	2443 (83%)
Flight times			
Median (IQR)	31 (20, 48)	55 (35, 88)	42 (25, 70)
Range	5–325	5–310	5–325
Time to hypotension during flight			
Median (IQR)	10 (4, 19)	15 (4, 33)	11.5 (4,25)
Range	0–143	0–160	0–160
Patient numbers (%)			
Total	1418	1509	2927
Hypotension	140 (10%)	170 (11%)	310 (11%)
During flight	121 (9%)	147 (10%)	268 (9%)
Outside flight	15 (1%)	13 (1%)	28 (1%)
Missing time^b^	4 (0%)	10 (1%)	14 (0%)
During flight among TBI	24 (31%)	83 (40%)	107 (37%)
During flight non‐TBI	97 (7%)	64 (5%)	161 (6%)

Abbreviations: IQR, interquartile range; MAP, mean arterial blood pressure; TBI, traumatic brain injury.

^a^ Only includes those with an isolated head injury and GCS < 14.

^b^ Not included in analysed data set.

The overall rate of in‐flight hypotension was 268 out of 2927 patients (9.2%). In primary retrievals, the rate was 8.5% % compared to 9.7% in IHTs. In non‐TBI cases, the rate was 6.1%. In contrast, TBI cases had much higher rates of in‐flight hypotension for both primary (30.8%) and IHTs (39.5%), with an overall rate of 37.2%.

**FIGURE 1:**
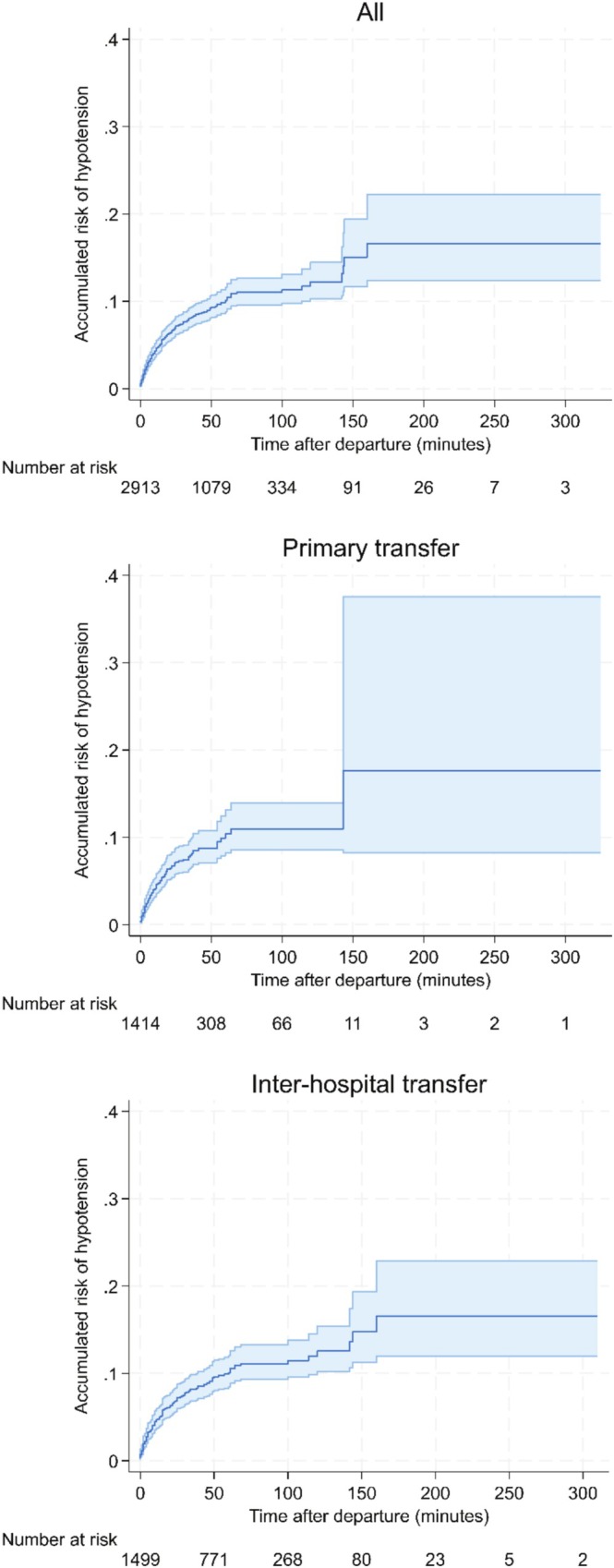
Accumulated risk of in‐flight hypotension over time by transfer type.

**FIGURE 2:**
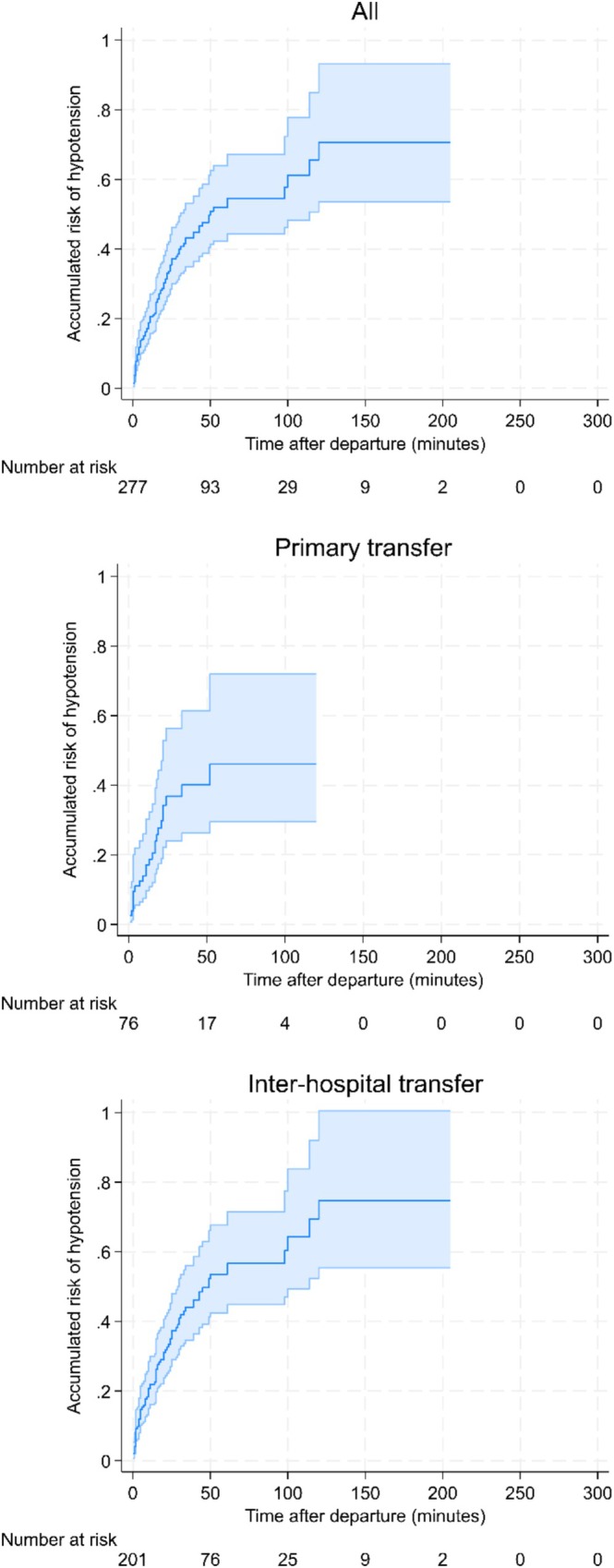
Accumulated risk of in‐flight hypotension among TBI cases over time by transfer type.

The risk of in‐flight deterioration was highest in the first 50 min, as demonstrated by the steep slope in Figure 1, following which the cumulative risk then gradually tapered. Past approximately 150 min, the small number of patients remaining at risk (i.e., in the air) results in wide confidence intervals, reflecting significant uncertainty. This relationship is consistent across the combined data, primary retrievals, and inter‐hospital retrievals. The relationship also persists when cases within 5 min of take‐off and landing are considered not to be hypotensive in a sensitivity analysis and when those continuing on vasopressors are excluded (see Figures S1 and S2). The total case numbers are smaller among TBI cases, resulting in wider uncertainty around estimates, yet it is clear that the accumulated risk is much higher among TBI cases. Again, hypotension occurred most frequently within the initial 30–50 min of flight (Figure 2).

## 4 Discussion

This paper presents the first evidence to confirm the physiologically plausible assumption that longer aeromedical transfer times are associated with an increased cumulative risk of deterioration during flight. Potential factors contributing to this include a longer time for clot disruption, coagulopathy, and ongoing bleeding resulting in haemodynamic compromise, a longer time for the effect of sedating medications to result in vasodilation in the intubated patient, and progression of underlying injuries, including pneumothorax [4, 5, 10]. As such, retrieval clinicians should consider the institution of interventions at a lower threshold in trauma patients undergoing long‐distance transfer, including prior to take‐off. To our knowledge, there are currently no other studies specifically examining this phase of care for trauma patients.

Interestingly, most of the clinically significant risk occurs early, particularly within the first 50 min. This pattern is observed in primary cases, IHTs, and TBI. Several factors may contribute to this. The associated altitude and barometric pressure changes after take‐off can have multiple clinical effects, particularly in non‐pressurised fixed‐wing aircraft. In rotary‐wing aircraft, the vibration associated with take‐off can theoretically increase the risk of clot disruption but also result in spurious measurements from oscillatory blood pressure recordings [10]. Notably, our sensitivity analysis examining cases within 5 min of take‐off and landing yielded similar results, suggesting genuine hypotension episodes occurring soon after departure. Given the shorter time to hypotension in primary cases and the relatively steep slope of the cumulative hazard curve, particularly for TBI (Figures 1 and 2), this may suggest an increased role for pre‐departure optimisation. Alternatively, it may also represent providers prioritising transport to definitive care in primary cases, acknowledging that hypotension may occur in‐flight, and compromising pre‐departure optimisation to undertake interventions in‐flight in the interest of overall patient care.

Initially, a dual definition of deterioration was considered, in line with our previous work [8, 9], which included hypotension and the institution of critical care interventions. Unfortunately, due to the nature of the clinical forms used for documentation throughout most of the study period, there was infrequent documentation of when the interventions were commenced. This prevented a time‐to‐event analysis. Fortunately, the clinical form utilised by LifeFlight retrieval teams has subsequently been amended to include a specific box to document intervention timings. As such, future work could benefit from including an analysis of when the first critical care intervention occurred in flight. This would also allow for further examination of the role of pre‐departure optimisation, particularly if many critical care interventions were occurring early in flight.

We deliberately chose to include only MAP as a measure of hypotension despite previously considering SBP as well. Notably, the specific patient monitor utilised by LifeFlight throughout the study period is oscillometric, directly measuring MAP and then calculating SBP via a proprietary algorithm [11]. Given this, for the sake of simplification and accuracy in meeting predetermined definitions, it was considered appropriate to consider only MAP.

The large difference observed in the percentage of hypotension in TBI as compared to general cases was unexpected. Notably, the definition of hypotension in TBI was a mean arterial pressure (MAP) of less than 80 mmHg, in line with the TBI literature, demonstrating that even isolated episodes of hypotension result in poorer neurological outcomes [12, 13]. Given the small total case numbers in our TBI cohort, it remains unclear why, in this group, where it would be reasonable to expect retrieval clinicians to more aggressively prevent hypotension, the observed rates were higher.

## 5 Limitations

This study was retrospective and included data from a single organisation, albeit one that conducts a wide variety of tasks. Furthermore, most cases (> 99%) were for blunt trauma. Therefore, this data may not be applicable to other jurisdictions, particularly those with different patient demographics or a higher proportion of penetrating trauma.

Recorded mission times were based on the actual time of departure and arrival at the receiving centre. The time that an aeromedical platform lands is not specifically documented in the database access. For rotary‐wing transfers, the time of arrival at the receiving centre and landing time are typically identical in Queensland, with the majority of accepting facilities having on‐site helipads. However, for a subset of rotary‐wing taskings and all fixed‐wing taskings, the transfer time will include a transfer from the landing site to the referring facility. Currently, there is no practical way to address this issue retrospectively.

Case inclusion relied on clinicians accurately submitting a relevant diagnosis at the end of their clinical notes. In the initial screening process, many cases with a diagnosis listed as trauma were excluded, as a non‐traumatic diagnosis would have been appropriate. It is unclear what proportion of actual trauma cases received a non‐trauma diagnosis and were therefore not included in the study. Given the focus on trauma in aeromedical retrieval, this likely represents a small number of cases, but there is a lack of robust data to quantify this group.

In Queensland, most clinically significant trauma cases undergoing aeromedical retrieval are managed by a LifeFlight medical doctor, with the major exception being several Royal Flying Doctor Service bases (not included in this study). As such, the included cases represent the bulk of the more severe trauma cohort with centrally stored clinical notes for reliable data access.

Finally, in our study, the primary outcome was binary—hypotension per definition or not. From a clinical perspective, persistent hypotension is likely of more interest to clinicians. This should be a consideration in further prospective work.

## 6 Conclusion

In this large retrospective case review, the duration of transfer is positively associated with the risk of in‐flight hypotension, with most of this risk occurring during the early stages of flight. These risks are similar in both primary and IHT retrievals. This expands on the prior knowledge base, providing insight into the risk of deterioration in a specific time period during a patient's journey that was not previously well‐characterised.

## Ethics Statement

The Darling Downs Hospital and Health Service Ethics Department approved this study—HREC/2022/QTDD/87421.

## Conflicts of Interest

The authors declare no conflicts of interest.

## Supporting information


**Figure S1:** Re‐analysis with episodes of hypotension within 5 min of take‐off or landing re‐classified as not meeting the definition for hypotension.

**Figure S2:** Sensitivity analysis two demonstrating cumulative risk curves with cases where vasopressors were commenced prior to flight excluded.

## Data Availability

The data that support the findings of this study are available from LifeFlight Medical. Restrictions apply to the availability of these data, which were used under license for this study. Data are available from the author(s) with the permission of LifeFlight Medical.
